# Specialised Competencies and Artificial Intelligence in Perioperative Care: Contributions Toward Safer Practice

**DOI:** 10.3390/healthcare13243286

**Published:** 2025-12-15

**Authors:** Sara Raposo, Miguel Mascarenhas, Ricardo Correia Bezerra, João Carlos Ferreira

**Affiliations:** 1 Hospital CUF Porto, 4100-180 Porto, Portugal; scraposo@hotmail.com; 2 Santa Maria Health School, 4049-024 Porto, Portugal; 3 Department of Community Medicine, Information and Health Decision Sciences (MEDCIDS), Faculty of Medicine, University of Porto, 4099-002 Porto, Portugal; 4 BioGHP, 4200-194 Porto, Portugal; 5 Faculty of Logistics, Molde University College, NO-6410 Molde, Norway; joao.carlos.ferreira@iscte-iul.pt; 6 ISTAR, ISCTE-Instituto Universitário de Lisboa, 1649-026 Lisboa, Portugal

**Keywords:** artificial intelligence, perioperative care, patient safety, clinical competencies, digital literacy, ethical implementation

## Abstract

This narrative review explores how specialised clinical competencies and artificial intelligence (AI) technologies converge in the context of perioperative care, with a focus on their combined potential to improve patient safety. Considering the growing complexity of surgical care and rising demands on healthcare professionals, the study aims to understand how human expertise and digital tools can complement each other in this high-stakes environment. Methods: A narrative review methodology was adopted to integrate insights from diverse sources, including empirical studies, policy documents, and expert analyses published over the last decade. Findings reveal that AI can support clinical decision-making, streamline workflows, and enable earlier identification of complications across all perioperative phases. These technologies enhance, rather than replace, the roles of nurses, anesthetists, and surgeons. However, their effective use depends on critical factors such as digital literacy, interdisciplinary collaboration, and ethical awareness. Issues related to data privacy, algorithmic bias, and unequal access to technology highlight the need for thoughtful, inclusive implementation. The future of perioperative care will likely depend on hybrid models where human judgment and AI-based tools are integrated in ways that uphold safety, equity, and person-centred values.

## 1. Introduction

Perioperative care, encompassing preoperative, intraoperative, and postoperative phases, is a high-stakes process where patient safety is paramount. The increasing complexity of surgical procedures, driven by an ageing population and rising multimorbidity, demands advanced clinical competencies from healthcare professionals, including nurses, anaesthetists, and surgeons. Artificial intelligence (AI), including machine learning, deep learning, and natural language processing, offers transformative potential by supporting clinical decision-making, optimizing workflows, and enhancing patient safety outcomes. This narrative review aims to answer the research question: What is theevidence on the impact of artificial intelligence on patient safety in perioperative care, and how does it integrate with specialised clinical competencies?

Artificial intelligence (AI) is rapidly transforming healthcare, offering significant potential to enhance safety and efficiency in complex medical procedures such as perioperative care [1,2]. By providing innovative solutions for monitoring, diagnosis, prediction, and management, AI mitigates risks associated with intricate care processes [3]. Advanced algorithms and machine learning enable analysis of vast datasets to generate real-time insights, optimize workflows, and support clinical decision-making [2]. This is particularly relevant in conditions such as sepsis, where early detection and personalized treatment strategies are critical, given its heterogeneous nature and variable progression [4,5]. AI’s ability to integrate diverse data, including medical history, symptoms, and imaging, facilitates early complication detection and personalized treatment planning [6].

In the perioperative continuum, AI extends beyond diagnostics to predictive capabilities for postoperative outcomes, enabling timely interventions that reduce adverse events [6]. At the institutional level, AI also identifies systemic inefficiencies and predicts potential harms, contributing to quality improvement initiatives [7]. Its cognitive functions: learning, reasoning, and problem-solving, support healthcare professionals by generating evidence-based recommendations, improving diagnostic accuracy, and enhancing therapeutic precision [8,9,10,11]. Within perioperative contexts, AI applications range from imaging-based early complication detection [12,13,14] to data-driven support for treatment planning, robotic-assisted surgeries, and workflow optimization [15,16,17,18]. Collectively, these tools strengthen perioperative safety and outcomes by enabling proactive and precise interventions [17,18].

The growing availability of healthcare data, coupled with rapid advances in analytical techniques, has accelerated AI’s integration into clinical practice [16]. AI systems excel at processing complex information with speed and precision beyond human capability, automating repetitive tasks while enabling clinicians to focus on complex decisions and direct patient care [19,20]. The exponential growth of data from electronic health records, wearable devices, and real-time physiological monitoring has created opportunities for AI-driven proactive health management and predictive interventions [21,22,23]. These advancements are particularly significant for perioperative care, where early complication recognition, optimized therapeutic compliance, and personalized care pathways can drastically improve safety and efficiency [24,25,26,27,28].

This review highlights the competencies clinicians need to safely and effectively integrate AI into perioperative care, including data interpretation, critical appraisal of AI-generated recommendations, and awareness of ethical and privacy concerns [17,29]. By focusing on competencies, it underscores the importance of aligning technological innovation with professional practice to ensure patient safety and equitable access [29,30]. It also emphasizes that AI is not a singular solution but a versatile toolset that augments perioperative safety through diagnostics, predictive analytics, and system-level improvements [30,31]. Ultimately, this synthesis advances the discussion by framing AI integration as both a clinical and safety imperative, while outlining the essential skills required to harness its full potential responsibly [21,25,32].

## 2. Materials and Methods

Perioperative care covers the full spectrum of healthcare services provided before, during, and after surgery. It is a critical process aimed at ensuring patient safety and improving surgical outcomes. This type of care must address both the physiological and psychological needs of individuals undergoing surgery, especially in high-acuity environments, where even minor errors can have serious consequences [32,33]. The global demographic shift towards an ageing population, coupled with a rise in multimorbidity, has made surgical cases more numerous and more complex [34,35]. As a result, perioperative teams are increasingly called upon to care for vulnerable patients with complex medical conditions. These evolving demands highlight the need for a workforce with advanced expertise, particularly among perioperative nurses, anaesthetists, and surgeons. Their roles require not only clinical skill and procedural know-how, but also strong situational awareness and effective collaboration across professions [36]. Alongside these trends, the healthcare field is rapidly incorporating artificial intelligence (AI). Only peer-reviewed, English-language studies published between 2015 and 2025 were included. Non-English sources and grey literature were excluded. This umbrella term includes techniques such as machine learning, deep learning, and natural language processing, tools that allow systems to learn from data and perform tasks traditionally carried out by humans [16,37]. In surgical settings, AI is starting to prove itself as a powerful support tool, offering benefits such as predictive analytics, improved workflow coordination, and enhanced precision in diagnosis and treatment [38,39]. This narrative review looks at how specialised perioperative competencies and AI technologies intersect. The aim is to shed light on how these elements can work together to improve patient safety, support evidence-based practices, and cultivate a culture of continuous improvement in surgery. It places particular emphasis on the complementary nature of human clinical skills and AI, including the use of predictive algorithms and intraoperative decision support tools such as chatbots and computer vision technologies [38,40], recognising that while human expertise remains indispensable, technology can significantly bolster decision-making and efficiency [41,42].

### 2.1. Research Question

The review addresses the question: What is the evidence on the impact of artificial intelligence on patient safety in perioperative care, and how does it integrate with specialised clinical competencies? This question focuses on AI applications that enhance the roles of clinicians and improve safety outcomes across perioperative phases.

### 2.2. Search Strategy

A narrative search was conducted in PubMed, Scopus, CINAHL, and Web of Science for studies published between January 2015 and June 2025. Search terms included combinations of:

Query 1: (“artificial intelligence” OR “AI” OR “machine learning”) AND (“perioperative care” OR “surgery” OR “anesthesia”) AND (“clinical competencies” OR “nursing” OR “surgeon”) AND (“patient safety”)

Query 2: ((MH “Artificial Intelligence” OR “artificial intelligence” OR “AI” OR “machine learning”) AND (MH “Perioperative Nursing” OR “perioperative care” OR “surgery” OR “anesthesia”) AND (MH “Clinical Competence” OR “nursing” OR “surgeon”) AND (MH “Patient Safety” OR “patient safety”))

Query 3: (“artificial intelligence” OR “machine learning”) AND (“perioperative care”) AND (“patient safety”) AND (“clinical competence” OR “clinical skill*” OR “professional competence” OR “nursing competence” OR “surgical competence” OR “anaesthetist” OR “surgeon” OR “nurse”)

Query 4: (“artificial intelligence” OR “machine learning”) AND (“perioperative care”) AND (“patient safety”).

Additional hand-searching of reference lists and grey literature was performed to identify relevant studies.

### 2.3. Inclusion and Exclusion Criteria

Inclusion Criteria: Peer-reviewed studies in English, published between 2015 and 2025, focusing on AI applications in perioperative care, their impact on patient safety, and their integration with clinical competencies of nurses, anaesthetists, or surgeons. Only full-text articles in English were eligible.

Exclusion Criteria: Studies not related to perioperative care, not involving AI, not addressing patient safety or clinical competencies, non-peer-reviewed articles, or studies in languages other than English.

Titles and abstracts were screened for relevance, followed by full-text review of potentially eligible studies. Two reviewers independently assessed studies for inclusion, resolving discrepancies through discussion.

This study is a narrative review with a structured search. We synthesized peer-reviewed evidence on AI in perioperative care with emphasis on specialized competencies. Because our objective was conceptual integration (competency frameworks, practice implications) rather than quantitative pooling, we did not follow PRISMA nor perform meta-analysis.

The initial database search yielded 3550 records. After applying filters for publication period (2015–2025), language (English), and full-text availability, 1363 articles remained for screening. Titles and abstracts were independently reviewed, and 59 studies met the inclusion criteria and were incorporated into the final narrative synthesis.

### 2.4. Quality Assessment

The quality of included studies was assessed using appropriate tools, such as the Cochrane Risk of Bias for randomized trials and the Newcastle–Ottawa Scale for observational studies. Systematic reviews were evaluated using the AMSTAR 2 checklist.

Due to heterogeneity in study designs and outcomes, a narrative synthesis was conducted, grouping findings by perioperative phase (preoperative, intraoperative, postoperative) and clinical role (nurses, anaesthetists, surgeons). Key themes included AI’s impact on patient safety, integration with clinical competencies, and implementation challenges.

All included evidence was mapped to perioperative phases (preoperative, intraoperative, postoperative) and to competency domains, enabling phase-specific synthesis and identification of gaps (Table S1; Figure S1).

## 3. Results

### 3.1. Specialised Competencies and Artificial Intelligence in Peri-Operative Care: Contributions Toward Safer Practice

Artificial intelligence (AI) is increasingly recognized for its potential to enhance peri-operative care, contributing to safer practices through various specialized competencies. We anchor our analysis in established perioperative competency frameworks, NOTSS (Situation Awareness, Decision Making, Communication & Teamwork, Leadership) and ANTS, (Task Management, Team Working, Situation Awareness, Decision Making) and examine how AI tools interact with, stress, or extend these domains, while also proposing two AI-augmented competencies, Data/AI Literacy and Human–AI Teaming, as necessary additions for safe practice.

Among included studies, 1/88 explicitly referenced NOTSS/ANTS. A further 3/88 measured outcomes that map implicitly to framework domains (e.g., response time and closed-loop communication as Teamwork, escalation thresholds as Decision Making). Alignment gaps were prominent in Leadership and Task Management where AI changes role allocation, but outcomes were seldom measured.

#### 3.1.1. Key Contributions of AI in Peri-Operative Care

Preoperative Optimization: AI can assist in preoperative risk stratification and prehabilitation, improving patient outcomes by identifying high-risk patients and optimizing their condition before surgery [43]. AI-driven predictive analytics can forecast perioperative risks such as adverse outcomes and the need for blood transfusions, enabling better preparation and planning [44]. Intraoperative Support: AI enhances intraoperative decision-making and technical skill augmentation, providing real-time imaging analysis, robotic assistance, and intraoperative monitoring to improve precision and minimize complications [45,46,47,48]. AI applications in surgery include semi-autonomous performance of tasks, technical skill assessment, and resource allocation, which collectively contribute to safer surgical practices [45,46]. Postoperative Care: AI aids in postoperative monitoring and management, enabling personalized recovery plans, early complication detection, and long-term followup [43,44,45,46]. Predictive analytics during postoperative care can tailor rehabilitation programs, improving recovery times and patient outcomes [48].

#### 3.1.2. Specialized Competencies Enhanced by AI

**Figure 1:**
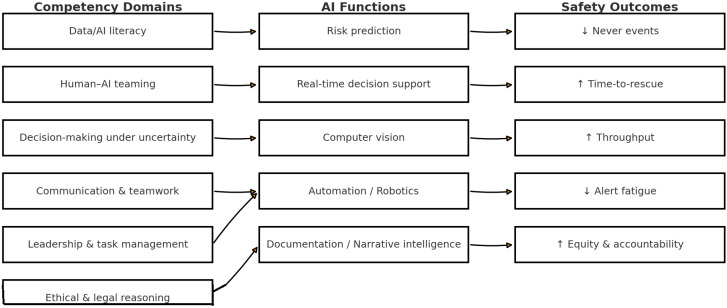
Competency–Function–Outcome map for AI-enabled care. Six competency domains (**left**) map to five AI functions (**middle**), which in turn are hypothesized to drive safety outcomes (**right**). Arrows indicate information/decision flow; **↑** denotes an increase/improvement (e.g., time-to-rescue, throughput, equity) and **↓** denotes a reduction (e.g., never events, alert fatigue).

We operationalize six competency domains (Table 1) and map them to AI functions (risk prediction, real-time decision support, computer vision, automation, documentation/narrative intelligence). For example, data/AI literacy enables safe thresholding of risk scores; team communication supports briefing/hand-over of AI signals; ethical reasoning governs override and escalation decisions. Table 1 Competencies × AI functions. Decision Support Systems: AI-driven decision support systems analyse complex datasets to provide actionable insights, improving clinical decision-making across the perioperative continuum [44,49,50]. Risk Prediction and Management: AI models predict perioperative risks and optimize patient management, addressing the increasing complexity of surgical patients and rising surgical volumes [44,48]. We operationalise six competency domains (Table 1) and map them to AI functions (risk prediction, real-time decision support, computer vision, automation, documentation/narrative intelligence). For example, data/AI literacy enables safe thresholding of risk scores; team communication supports briefing/hand-over of AI signals; ethical reasoning governs override and escalation decisions. Technical Skill Augmentation: AI technologies such as convolutional neural networks (CNNs) and support vector machines (SVMs) enhance diagnostic precision and surgical planning, aiding in tasks like fracture detection and osteoarthritis grading [48,51]. To operationalize this taxonomy for practice and measurement, we separate technical competencies (device operation, data validation, interpreting model outputs) from non-technical competencies (decision-making under uncertainty, communication, leadership, ethics) and map them to perioperative phases. Figure 1 synthesizes the competency–AI–safety framework, linking the six domains (left) to AI functions (center) and safety outcomes (right), providing the scaffold for phase-specific operationalization (Table 2).

#### 3.1.3. Challenges and Considerations

Ethical and Legal Issues: The integration of AI in perioperative care raises ethical and legal concerns, including algorithm transparency, data standardization, and accountability in high-stakes scenarios [44,47,52].

Implementation and Collaboration: Successful AI implementation requires collaboration between surgeons, anesthesiologists, and computer scientists, along with robust safety protocols and prospective clinical trials [47,52].

AI holds significant promise in transforming perioperative care by enhancing preoperative planning, intraoperative precision, and postoperative management. While challenges remain, ongoing research, innovation, and interdisciplinary collaboration are essential to fully realize AI’s potential in improving patient safety and outcomes in perioperative medicine [43,44,45,46,49,50,51,52].

### 3.2. Enhancing Patient Safety in Peri-Operative CareThroughAI 

To enhance patient safety in peri-operative care through artificial intelligence (AI), several key areas have been identified where AI can make significant contributions. 

#### 3.2.1. Preoperative Risk Assessment and Planning 

Risk Stratification: AI systems can predict perioperative risks by analyzing patient data, which helps in identifying patients at increased surgical risk and planning appropriate interventions [53,54,55]. Preoperative Optimization: AI-driven tools assist in preoperative assessments, optimizing patient conditions before surgery to reduce complications [53,56]. 

#### 3.2.2. Intraoperative Management 

Real-time Decision Support: AI provides real-time support during surgeries by predicting adverse events such as hypotension or hypoxemia, thus allowing for timely interventions [55,57]. Precision and Control: AI-enhanced robotic systems improve surgical precision, reduce complications, and enhance overall surgical outcomes [57,58,59,60,61]. 

#### 3.2.3. Postoperative Monitoring and Follow-Up 

Predictive Analytics: AI models predict postoperative complications, enabling early interventions and personalized recovery plans [59,60,61]. Remote Monitoring: Integration of AI with wearable technology and remote monitoring systems ensures continuous patient monitoring, improving safety and outcomes after discharge [46,56]. 

#### 3.2.4. Enhancing Efficiency and Reducing Errors 

Resource Optimization: AI optimizes operating room scheduling and resource allocation, reducing delays and improving efficiency [44,62]. Error Reduction: AI systems help in reducing medication and diagnostic errors by providing accurate and timely information [47,63]. 

#### 3.2.5. Ethical and Practical Considerations 

Ethical Challenges: The implementation of AI must address ethical concerns such as data privacy, algorithmic bias, and the need for transparent decision-making processes [55,62,64]. HumanAI Collaboration: AI is designed to augment rather than replace human expertise, ensuring that clinical judgment remains central to patient care [37,58]. In conclusion, AI has the potential to significantly enhance patient safety in peri-operative care by improving risk assessments, providing real-time support during surgeries, optimizing postoperative monitoring, and reducing errors, see Table 3. However, successful implementation requires addressing ethical challenges, ensuring data security, and fostering collaboration between AI systems and healthcare professionals [53,54,55,56,57,58,59]. 

#### 3.2.6. Robotic and Computer-Assisted Surgery 

AI augments robotic and computer-assisted surgery through intraoperative scene understanding (tool/tissue tracking), context-aware assistance (automated camera repositioning, tremor filtering), and objective skill assessment from kinematic/vision streams. In preoperative planning, AI-driven segmentation and patient-specific models inform port placement and risk mapping; intraoperatively, real-time classifiers support safe dissection planes and anticipate adverse events (e.g., bleeding), while postoperatively trajectory features predict complications and guide targeted remediation. These capabilities remain assistive (Levels 0–2 autonomy) and operate under surgeon oversight.

### 3.3. Stakeholders

Perioperative care is, by its very nature, a profoundly collaborative endeavour, drawing upon the coordinated expertise of diverse healthcare professionals whose distinct yet complementary roles converge in the shared mission of safeguarding patient wellbeing. Nowhere is this interdependence more evident than in the high-pressure, rapidly evolving environment of surgical care, where clinical competence must be matched by adaptability, trust, and clear communication [65].

Among the many contributors to this intricate process, nurses stand as central figures throughout the entire surgical journey. Their involvement spans from the initial stages of patient preparation, through education, psychological support, and clinical assessment, to vigilant intraoperative monitoring and dedicated post-operative care. Perioperative nurses play a pivotal role in maintaining patient safety and team coordination throughout the perioperative pathway [36,66,67]. Their competencies encompass not only technical skills such as infection prevention and early recognition of clinical deterioration, but also a human responsiveness that allows them to act swiftly and with compassion during unexpected events in the operating theatre [65,68]. The ability to communicate effectively, remain adaptable under pressure, and make rapid, ethically informed decisions is vital. As patient advocates, nurses ensure that clinical decisions remain grounded in ethical principles and individual needs. In critical moments, such as the onset of haemorrhage or anaesthetic complications, their capacity to detect subtle warning signs and intervene decisively can make the difference between stability and crisis [63,69].

In close partnership with nurses, anaesthetists carry the responsibility of maintaining the patient’s physiological equilibrium throughout surgery. Their role demands continuous and precise monitoring of cardiovascular, respiratory, and neurological parameters, grounded in a deep understanding of pharmacology and pathophysiology. Expertise in drug titration, trend interpretation, and proactive risk management is fundamental [19,29].

Yet beyond technical mastery, anaesthetists must possess the cognitive agility to adjust plans dynamically, especially in response to real-time changes. Increasingly, this includes the capacity to integrate data from AI-assisted monitoring systems, balancing algorithmic predictions with nuanced clinical judgement to refine anaesthetic management and optimise patient safety [66]. The fusion of human insight with technological tools reflects a shift in the profession, requiring both openness to innovation and a firm grounding in clinical reasoning.

Surgeons, at the heart of the operative process, must unite exceptional manual skill with strategic thinking and ethical discernment. Their tasks extend beyond the physical act of surgery to include critical decision-making in moments of profound uncertainty, such as when confronted with an unanticipated malignancy or complex vascular injury [70]. These decisions demand not only clinical precision but emotional intelligence, as surgeons must weigh risks and outcomes with urgency and clarity. Leadership is equally indispensable. The surgeon sets the tone in the operating theatre, coordinating multidisciplinary input, fostering calm amidst tension, and guiding collective responses to intraoperative challenges. The quality of this leadership can significantly influence both procedural success and the overall coherence of team dynamics [70,71].

What ultimately underpins safe perioperative care is not only individual excellence but effective interprofessional teamwork. A shared mental model, supported by structured communication strategies such as preoperative briefings and checklists, enhances situational awareness and reduces the likelihood of error. Tools like the WHO Surgical Safety Checklist have brought tangible improvements to surgical safety by standardising protocols and reinforcing a culture of mutual respect [5,29]. Beyond technical knowledge, it is this culture of collaboration that fortifies the perioperative environment. Interdisciplinary training initiatives contribute meaningfully to team cohesion and role clarity, especially during high-stakes scenarios, enabling smoother transitions, accurate documentation, and timely interventions [72]. The choreography of perioperative care, when executed well, is a testament to what can be achieved when professionals from distinct disciplines work with mutual trust and shared purpose. Central to the development and refinement of these competencies is simulation-based training. High-fidelity simulations recreate the complexities of real-life clinical situations, allowing teams to hone both procedural skills and interpersonal dynamics under realistic conditions. These scenarios foster confidence in managing crises, promote reflective practice, and strengthen communication under pressure [69,73,74]. Recent innovations using virtual reality and AI-enhanced simulation environments further support perioperative education and situational awareness [73]. Moreover, as surgical environments become increasingly shaped by digital technologies, the importance of lifelong learning becomes ever more evident. Clinicians must now cultivate digital fluency, not only to navigate new tools, but to critically engage with algorithmic outputs and integrate them meaningfully into patient care. In this evolving landscape, professional excellence depends not just on technical proficiency, but on a willingness to learn continuously, collaborate openly, and respond with both intellect and empathy to the unpredictable demands of surgical care [73,74]. Emerging hybrid roles, such as clinical informatics specialists and AIimplementation leads, are increasingly vital in bridging the gap between developers and clinical teams [75].

#### Simulation and AI-Readiness in Perioperative Teams

We define clinical simulation as the deliberate, fidelity-graded recreation of perioperative scenarios to practice technical and non-technical skills, debrief safely, and generate shared mental models. For safety-critical competencies (airway crisis, hemorrhage control, anaphylaxis), simulation measurably improves time-to-intervention and closed-loop communication. With AI entering OR workflows, simulation becomes the sandbox to (i) calibrate trust in alerts, (ii) practice handovers that incorporate AI outputs, and (iii) identify failure modes (alarm flooding, misclassification) before live use.

### 3.4. Artificial Intelligence General Overview

Artificial intelligence (AI) transforms perioperative care by augmenting the expertise of nurses, anaesthetists, and surgeons, fostering a collaborative model that enhances patient safety, precision, and efficiency across the surgical continuum. Far from replacing human clinicians, AI serves as a powerful ally, processing vast datasets, detecting subtle trends, and relieving cognitive burdens, while human judgment, ethical sensitivity, and contextual understanding remain indispensable. This synergy creates a responsive, person-centered approach to care, leveraging the strengths of both technology and clinical expertise. In the preoperative phase, AI systems analyze electronic health records to synthesize patient data, identifying those at risk for complications such as postoperative delirium, cardiovascular events, surgical site infections [75]. Predictive models provide probabilistic assessments of critical outcomes, such as postoperative mortality or intensive care needs, enabling tailored planning and informed consent discussions [6,19]. These tools enhance clinicians’ ability to make proactive decisions, complementing their expertise by offering a clearer picture of patient vulnerability. However, clinicians must interpret these outputs contextually, discerning whether, for example, a rising heart rate signals bleeding, anxiety, or pain, ensuring that AI recommendations align with the patient’s broader history and preferences [76]. Intraoperatively, AI contributes through technologies like computer vision and real-time analytics, which track instruments, recognize anatomical landmarks, and optimize fluid or drug delivery. AI-assisted anaesthesia platforms, equipped with smart alarms and adaptive dosing algorithms, maintain patient stability while reducing the mental workload of anaesthetists [77]. These systems excel at continuous, high-resolution monitoring, detecting physiological changes that may elude human observation, such as early signs of haemodynamic instability [76]. Yet, their effectiveness hinges on human oversight. Clinicians balance algorithmic predictions with nuanced judgment, ensuring that interventions reflect the patient’s unique clinical and personal context. For instance, decision support systems consolidate clinical data into intuitive dashboards, enabling evidence-based choices without undermining professional autonomy [78]. Postoperatively, AI enhances recovery surveillance by processing vital signs, laboratory parameters, and clinical notes to detect early indicators of deterioration, often beforethey are clinically evident [76]. Machine learning models shape individualized recovery plans, aligning post-discharge strategies with each patient’s needs, thus supporting a more anticipatory and humane care model. Beyond direct clinical applications, AI improves operational efficiency by optimizing surgical scheduling, anticipating resource needs, and reducing delays, which enhances patient experience and staff wellbeing [78]. These contributions demonstrate AI’s role as a versatile toolset, addressing both clinical and logistical challenges in perioperative care. The success of AIhuman collaboration depends on trust, transparency, and thoughtful integration. Clinicians must feel confident in the reliability and interpretability of AI outputs, as the “black box” phenomenon—where algorithms provide recommendations without clear explanations—can erode trust [79,80,81]. Explainable AI, which delivers outputs in a clinically understandable manner, is essential to foster meaningful adoption. Additionally, excessive or poorly calibrated alerts risk desensitizing clinicians, leading to alert fatigue and potential oversight of critical warnings. Interfaces must be intuitive, customizable, and aligned with clinical workflows to mitigate this risk [80]. Digital literacy is thus emerging as a core competency, requiring clinicians to understand how AI systems function, assess their limitations, and integrate outputs responsibly [79]. New hybrid roles, such as clinical informatics specialists, bridge the gap between developers and clinical teams, ensuring technologies meet real-world needs [82]. Despite its promise, AI introduces risks that must be addressed to ensure safe collaboration. Biases in training datasets, particularly when underrepresented groups are inadequately included, can lead to inaccurate predictions, reinforcing health disparities [80]. Many AI systems are validated in idealized settings, which may not reflect the complexity of real-world practice, increasing the risk of misapplication or over-reliance. Clinicians must apply critical judgment and moral reasoning to safeguard patient safety and dignity. Accountability remains a critical issue: when errors occur, determining responsibility—whether with the clinician, institution, or developer—requires clear legal and ethical frameworks [82]. Technical barriers, such as interoperability and cybersecurity, further complicate integration, necessitating robust regulation and infrastructure [79,82]. Ultimately, AIhuman collaboration in perioperative care amplifies the capacity to deliver precise, efficient, and compassionate care. By combining AI’s analytical prowess with clinicians’ empathy and expertise, this partnership fosters a safety-conscious culture, enhances team coordination through tools like AI-enhanced briefings, and promotes shared mental models [78]. The goal is not to supplant human expertise but to create intelligent partnerships that prioritize patient dignity and clinical equity. Achieving this vision requires interdisciplinary collaboration, transparent system design, and a commitment to aligning technology with the core values of perioperative care.

### 3.5. Implications for Clinical Practice and Professional Education

The growing integration of artificial intelligence into perioperative care is not only transforming clinical workflows but also redefining what it means to be a healthcare professional. As AI systems become increasingly embedded in surgical practice, they are changing both how clinicians work and what competencies they need to provide care that remains safe, effective, and humane. This shift has significant implications, not just for individual practitioners, but for healthcare institutions, education systems, and the broader structures that support professional development. At the forefront of these changes is the urgent need to expand existing competency frameworks to include digital and AI literacy. Clinicians are now expected to critically engage with technologies once limited to data scientists. It is no longer enough to use AI tools passively; professionals must understand how algorithms are developed, validated, and applied in clinical settings. Equally important is the ability to assess the reliability, transparency, and clinical relevance of AI-generated outputs [83]. This shift demands multidisciplinary training that bridges medicine, ethics, informatics, and data science. Academic institutions and regulatory bodies must work together to develop curricula that equip clinicians to use AI responsibly and question it thoughtfully and ethically [9]. However, education alone is not enough. Safe AI implementation also requires organisational adaptation. Institutional readiness involves not just technical infrastructure, but also strong governance to oversee AI use, maintain cybersecurity, and assess clinical outcomes. Leadership must foster a culture of curiosity, adaptability, and ethical awareness. This includes clarifying the purpose of AI tools, assigning accountability, and enabling open discussion of concerns and uncertainties [7]. Ethical frameworks and clear protocols should guide AI integration to ensure that tools prioritise patient care above efficiency. Ongoing professional development is essential for sustained technical and cultural change [78]. Crucially, AI tools must be designed with and for their users. Successful integration depends not just on technical sophistication but on practical relevance. Co-creation, involving clinicians in the design, testing, and refinement of AI systems, ensures alignment with real-world workflows and values [78]. Participatory design builds trust, improves usability, and reduces resistance, while top-down approaches that ignore frontline experience risk failure and harm. Early engagement ensures systems remain intuitive and respectful of clinical complexity [84]. Adapting clinical practice and professional education to AI is not merely technical, it is ethical. What is at stake is the preservation of core healthcare values: safety, compassion, responsibility, and human dignity. AI can offer significant benefits, enhancing clinical insight, efficiency, and outcomes, but only when guided by human judgement and aligned with real care contexts. The future of perioperative medicine will be most safely and meaningfully shaped through a balance of innovation with empathy, and precision with perspective.

### 3.6. Ethical, Legal, and Equity Considerations

As artificial intelligence becomes increasingly embedded in perioperative care, its potential to enhance safety, precision, and efficiency is accompanied by a growing set of ethical, legal, and equity-related concerns. The way these technologies are developed, implemented, and governed will profoundly shape their impact, not only on individual patients, but on the wider values and structures of healthcare. Ensuring that AI serves the goals of fairness, transparency, and accountability is therefore essential, both to protect those receiving care and to maintain the integrity of those delivering it. At the heart of these concerns lies the question of data. AI systems rely heavily on vast datasets, frequently drawn from electronic health records or perioperative registries, to train algorithms and refine their performance. While this data holds immense potential for improving clinical insight and patient outcomes, its secondary use raises important issues around privacy, consent, and data governance. Patients have the right to understand not only how their personal health information is stored, but also how it may be used to build or operate AI tools that influence their care [83]. Respecting these rights demands more than passive compliance; it requires proactive communication, clear consent processes, and a commitment to digital dignity, where individuals remain informed and empowered regarding the fate of their own data [9]. Alongside concerns around data use, the legal and professional implications of AI-supported decision-making remain complex and, in many cases, unresolved. One of the most persistent challenges is that of accountability. When clinical decisions are influenced, directly or indirectly, by algorithmic outputs, and adverse outcomes occur, determining where responsibility lies is far from straightforward. Is it the clinician who acted on theAI’s recommendation? The institution that deployed the system? Or the developer who built it? In the absence of clear legal frameworks, such questions can undermine both trust in technology and confidence in its safe use. To preserve the ethical foundations of healthcare, it is essential that clinicians retain ultimate responsibility for the decisions made in the care of their patients [82]. Safe perioperative AI requires (1) pre-deployment fairness audits with intersectional reporting; (2) alarm stewardship policies to manage alert fatigue; (3) clear override/appeal pathways and documentation in the record; and (4) post-deployment monitoring for drift and harm signals, with versioning and change control recorded as part of the device file. AI must be seen as an adjunct to, not a substitute for, clinical judgement, one that supports reflection, but never replaces moral reasoning or human accountability [70]. Another critical concern is equity. Without deliberate oversight, AI tools risk reinforcing or even exacerbating existing disparities in healthcare delivery. Biases embedded in training datasets, particularly when they fail to adequately represent minority populations, older adults, or individuals with complex or rare conditions, can lead to models that are less accurate, less effective, or even harmful for those groups [80,83]. These unintended consequences can deepen structural inequalities, especially if such systems are deployed uncritically or without sufficient validation across diverse populations. Research has documented algorithmic bias that directly affects care prioritisation, such as underestimating condition severity in black or older patients [76,80]. Addressing this requires a commitment to inclusive data practices from the earliest stages of AI development, including the systematic collection of diverse and representative datasets, and the ongoing evaluation of algorithmic performance across demographic lines. In addition, access to AI-enabled technologies is far from evenly distributed. Many healthcare institutions, particularly those in under-resourced settings, may lack the infrastructure, funding, or technical capacity to implement and sustain these innovations. As AI becomes increasingly integral to perioperative care, there is a real danger that a digital divide will emerge, leaving certain patient populations behind [84]. Promoting digital inclusion must therefore be a priority. This involves not only investing in scalable and affordable tools but also ensuring that healthcare workers in underserved areas receive adequate training and support to engage with them meaningfully. Policy efforts should be explicitly designed to reduce, not widen, inequalities in access to high-quality, data-driven care. Engaging with these ethical, legal, and equity considerations is not an optional supplement to technical innovation, it is a necessary foundation for responsible practice. Only by addressing questions of privacy, consent, fairness, and accountability head-on can we ensure that AI strengthens rather than fragments the moral fabric of healthcare. The aim must be not simply to create smarter tools, but to cultivate a system that is as just, inclusive, and compassionate as it is efficient. In doing so, artificial intelligence can be guided to serve its highest purpose: supporting care that is not only more precise, but also more human. 

### 3.7. Limitations of AI in Perioperative Care 

The safe use of AI in the perioperative setting requires a critical appraisal of its limitations, including dataset shift, bias, alert fatigue, explainability, liability, workflow fit, integration costs, and external validity. To address these challenges, mitigation strategies such as prospective trials, fairness audits, human-factors co-design, and post-deployment monitoring are essential. Within this framework, safe practice requires: (1) pre-deployment fairness audits with intersectional reporting; (2) alarm stewardship policies to manage alert fatigue; (3) clear override and appeal pathways with documentation in the clinical record; and (4) post-deployment monitoring to detect drift and harm signals, with versioning and change control recorded as part of the device file. 

### 3.8. Research Gaps and Future Directions 

Despite growing evidence, significant research gaps remain. Most studies to date are retrospective or single-centre, with limited prospective, multi-site evaluations capable of establishing external validity and cost-effectiveness. Equity auditing is insufficiently developed, particularly across intersectional demographics such as age, sex, ethnicity, and comorbidity profiles, raising the risk of amplifying disparities. While technical advances in predictive analytics, computer vision, and decision support are promising, their safe integration into perioperative workflows requires simulation-based human-factors research to optimise trust calibration, alarm management, and override behaviours. Governance and accountability frameworks also lag behind technological progress, leaving medico-legal responsibility unresolved in high-stakes scenarios. Furthermore, robust post-deployment monitoring for dataset drift, emergent harms, and unintended behavioural effects remains rare. Addressing these gaps through multicentre trials, systematic fairness audits, and transparent regulatory standards will be essential for AI to evolve from experimental innovation to a trusted partner in safe, equitable, and patient-centred perioperative care. 

## 4. Conclusions

This narrative review has highlighted that, while artificial intelligence introduces significant innovation to perioperative care, it is ultimately the synthesis of technology with specialised human expertise that drives safer and more effective outcomes. The experience, ethical awareness, and relational skills of perioperative nurses, anesthetists, and surgeons remain indispensable, particularly when managing the inherent unpredictability and emotional complexity of surgical care. When implemented thoughtfully and ethically, AI has the potential to enhance these clinical strengths by improving precision, streamlining operations, and supporting real-time decision-making, thus shifting the focus from reactive measures to proactive patient safety. In this emerging landscape, the integration of predictive algorithms, smart monitoring systems, and decision support tools can prove to be valuable allies, provided they are trustworthy, transparent, and finely tuned to clinical realities. Looking ahead, it is anticipated that the next generation of perioperative care will be defined by hybrid models in which human expertise and digital support evolve together. Realising this vision will require substantial investment not only in infrastructure but also in professional education, governance, and participatory innovation. Clinicians must be recognised not merely as end-users of AI, but as co-designers and stewards, thereby ensuring that new tools remain aligned with the core values of person-centred care [80,81,82]. Priority gaps include prospective, multi-site evaluations of AI-augmented perioperative bundles; equity auditing against intersectional demographics; and simulation-based human-factors trials to calibrate trust, alarm load, and override behaviors. Post-deployment monitoring frameworks (drift, harm signals) are needed to sustain safety benefits. As AI continues to expand its footprint within healthcare systems, the emphasis must remain steadfastly on ethical integrity, inclusiveness, and patient safety. A collaborative approach that brings together clinicians, developers, ethicists, and policymakers will be essential for constructing frameworks that are both robust and fair. Rather than being seen as a cure-all, AI should be regarded as a catalyst, one which, when applied responsibly, can transform surgical environments to be not only more intelligent but also profoundly more humane. Healthcare systems and professional organisations must therefore prioritise the integration of AI literacy into training programmes, actively support collaboration in digital health development, and rigorously test AI tools prior to their clinical deployment. By aligning human capability with technological progress, a perioperative care system that is simultaneously smarter, more efficient, and more compassionate, inclusive, and safe for all can be realised. Ongoing interdisciplinary collaboration between healthcare professionals, developers, and regulatory bodies will be essential to ensure that AI develops as a tool for clinical equity rather than a driver of exclusion [83,85].

## Figures and Tables

**Table 1:** Competencies × AI functions.

Competency Domain	Risk Prediction	Real-Time Decision Support	Computer Vision	Automation/Robotics	Documentation/Narrative Intelligence
Data/AI literacy	✓	✓	•	•	✓
Clinical reasoning & decision-making	✓	✓	•	•	•
Team communication & collaboration	•	✓	•	•	✓
Ethical & legal reasoning (governance)	•	✓	•	✓	✓
Digital workflow & informatics	•	•	✓	✓	✓
Safety, quality & human factors	•	✓	✓	✓	✓

**Table 2:** Technical vs. Non-technical Competencies by Perioperative Phase.

Phase	Technical (Examples)	Non-Technical (Examples)
Preoperative	Device setup; data validation; EHR feature checks; threshold tuning; consent capture	Shared decision-making; briefing with AI risks; leadership of preop huddles; ethics of consent
Intraoperative	Monitor/device operation; model output interpretation; CV overlays; override execution	Communication under time pressure; closed-loop on alerts; leadership & role clarity; ethical escalation
Postoperative	Remote monitoring tools; trend analysis; note generation QA; discharge risk scoring	Handover quality; team situational awareness during rescue; family communication; governance of follow-up

**Table 3:** Summary of AI Applications in Peri-Operative Care.

Application	Benefits	Challenges
Preoperative Risk Assessment	Improved risk stratification and patient optimization	Data integration and accessibility
Intraoperative Management	Real-time decision support, enhanced precision, and control	Algorithmic transparency and bias
Postoperative Monitoring	Early complication detection, personalized recovery plans, remote monitoring	Data privacy and security
Efficiency and Error Reduction	Optimized scheduling, reduced errors	Integration with existing systems
Ethical Considerations	Ensuring patient safety and ethical AI governance	Addressing ethical and legal concerns

## Data Availability

No new data were created or analyzed in this study.

## Supplementary Materials

The following supporting information can be downloaded at: https://www.mdpi.com/article/10.3390/healthcare13243286/s1. Table S1: Mapping of Included Studies; Figure S1: Distribution of Studies.
