# Supplementary material for: Specialised Competencies and Artificial Intelligence in Perioperative Care: Contributions Toward Safer Practice
Source: Healthcare (Basel). 2025 Dec 15;13(24):3286. doi: 10.3390/healthcare13243286 (PMC12733068; doi:10.3390/healthcare13243286)
Supplement: Supplementary file 1 [file healthcare-13-03286-s001.zip › healthcare-3871953-supplementary.pdf]

Supplementary Materials

Table S1. Mapping of Included Studies

First Author, Year	Study Design	Perioperative Phase	Clinical Role Focus	AI Functions	Competency Domains	Safety Outcomes
Lee, 2018 [39]	Retrospective cohort	Pre/Post	Anaesthetists, Surgeons	RP, DS	DAI, CRD, SQH	Mortality prediction
Maheshwari, 2023 [46]	Review	Multi	Anaesthetists	RP, DS, OM	DAI, CRD, DWI	Workflow efficiency
Chevalier, 2025 [45]	Systematic Review	Intra	Surgeons	CV, AR	DAI, CRD, SQH	Precision & complications

Figure S1. Distribution of Studies

Figure S1 presents a heatmap visualizing the distribution of the 59 included studies across the intersection of perioperative phases and clinical competency domains.

The Y-axis lists the four perioperative phases used in the review: 1) Preoperative; 2) Intraoperative; 3) Postoperative; and 4) Multi-phase (studies covering ≥2 phases)

The X-axis lists the six competency domains from the competency framework used in the manuscript: 1) Data/AI literacy; 2) Clinical reasoning and decision-making; 3) Team communication and collaboration; 4) Ethical and legal reasoning (governance); 5) Digital workflow and informatics; and 6) Safety, quality, and human factors.

Each cell of the heatmap displays:

- the number of studies addressing the specific combination of perioperative phase and competency domain, and
- a color intensity proportional to the relative evidence density (darker = more studies).

The figure highlights areas with strong evidence concentration—particularly intraoperative studies focused on clinical reasoning, safety/human factors, and real-time AI-enabled decision support—as well as areas with clear research gaps, such as the ethical/legal domain and team communication, especially in the preoperative and postoperative phases.

Figure S1 shows a heatmap mapping perioperative phases to competency domains.

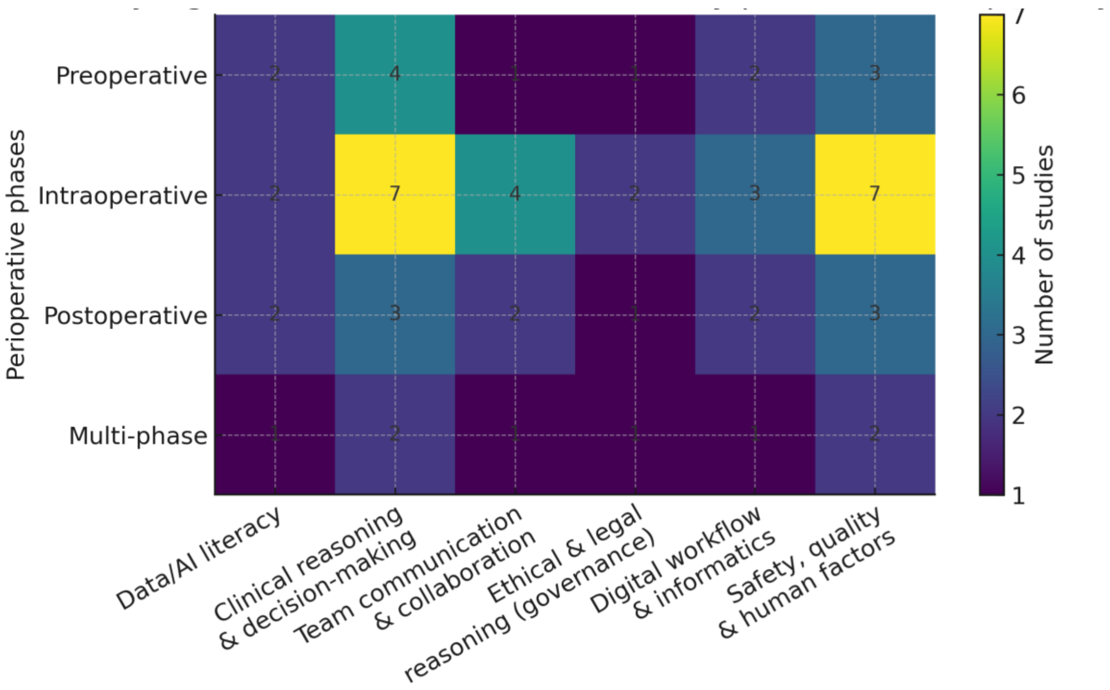

Figure S1. Heatmap illustrating the distribution of the 59 included studies across perioperative phases (Y-axis) and clinical competency domains (X-axis). Each cell shows the number of studies addressing a given combination, with colour intensity proportional to evidence density. The mapping reveals strong evidence clusters in intraoperative clinical reasoning, safety, and human-factors-related competencies, while identifying gaps in ethical/legal governance and team communication across several perioperative phases. Study-level details corresponding to each cell are provided in Supplementary Table S1.
